# Feasibility and optimal choice of stimulation parameters for supramaximal stimulation of motor evoked potentials

**DOI:** 10.1007/s10877-022-00972-5

**Published:** 2023-01-13

**Authors:** S. E. Dulfer, F. Lange, M. M. Sahinovic, F. H. Wapstra, A. R. Absalom, C. Faber, R. J.M. Groen, G. Drost

**Affiliations:** 1 Department of Neurosurgery, University Medical Center Groningen, University of Groningen, Groningen, The Netherlands; 2 Department of Neurology, University Medical Center Groningen, University of Groningen, Groningen, The Netherlands; 3 Department of Anesthesiology, University Medical Center Groningen, University of Groningen, Groningen, The Netherlands; 4 Department of Orthopedics, University Medical Center Groningen, University of Groningen, Groningen, The Netherlands; 5 Hanzeplein 1, 9713 GZ Groningen, The Netherlands

**Keywords:** Intraoperative neurophysiological monitoring, Scoliosis surgery, Transcranial electrical stimulation motor evoked potentials, Stimulation settings, Interstimulus interval, Pulse duration

## Abstract

**Purpose:** The aim was to investigate the feasibility and optimal stimulation parameters for supramaximal stimulation of muscle recorded transcranial electrical stimulation motor evoked potentials (mTc-MEP). **Methods:** Forty-seven consecutive patients that underwent scoliosis surgery were included. First, the feasibility of supramaximal stimulation was assessed for two settings (setting 1: pulse duration 0.075ms, interstimulus interval (ISI) 1.5ms; setting 2: pulse duration 0.300ms, ISI 3ms). Thereafter, three mTc-MEP parameters were considered for both settings; (1) elicitability, (2) amplitude, and (3) if supramaximal stimulation was achieved with ≥ 20 V below maximum output. Finally, ISIs (1ms–4ms) were optimized for setting 1. **Results:** Nine patients (19.15%) were excluded. Of the remaining patients, supramaximal stimulation was achieved in all patients for setting 1, and in 26 (68.42%) for setting 2. In one patient, mTc-MEPs were elicitable in more muscles for setting (1) Amplitudes were not significantly different. Stimulation voltage could be increased ≥ 20 V in all 38 patients for setting 1 and in 10 (38.46%) for setting (2) Optimal ISI’s differed widely. **Conclusion:** We recommend using setting 1 when monitoring mTc-MEPs with supramaximal stimulation, after which an individualized ISI optimization can be performed. Moreover, when using supramaximal stimulation, short ISI’s (i.e. 1ms or 1.5ms) can be the optimal ISI for obtaining the highest mTc-MEP amplitude.

**Supplementary Information:**

The online version contains supplementary material available at 10.1007/s10877-022-00972-5.

## Introduction

For the past three decades, intraoperative neurophysiological monitoring (IONM) has been used to detect and prevent neurologic injury during high-risk spinal surgery [1, 2]. The sensory tracts can be monitored using somatosensory evoked potentials (SSEPs) [3], while the integrity of motor tracts can be determined using muscle recorded transcranial electrical stimulation motor evoked potentials (mTc-MEPs) [4]. The combined use of SSEPs and mTc-MEPs has become an essential tool for detecting and preventing surgically induced neurological injury during scoliosis surgery [1, 5].

Various mTc-MEP monitoring methods and corresponding warning criteria can be used to determine impending neurological damage. Three main methods have been described. These are: the threshold level method [6, 7], the waveform method [8], and the amplitude reduction method [9]. Langeloo et al. concluded that, even though all three methods can successfully be used for monitoring, the amplitude reduction method is the most sensitive predictor of neurological damage during spinal surgery [10]. Depending on the type of surgery, a decrease of 50 to 100% from the baseline mTc-MEP amplitude is considered a warning [4, 11, 12].

The amplitude reduction method can be applied using either submaximal stimulation or supramaximal stimulation. In submaximal stimulation, the stimulation voltage is usually increased to 20–30% above the threshold stimulation voltage [13]. In supramaximal stimulation, the voltage is increased beyond the voltage necessary to evoke the maximum amplitude of the mTc-MEPs [10, 14]. An advantage of performing supramaximal stimulation is that it yields less variability between consecutive mTc-MEP amplitudes than submaximal stimulation, as has been reported in a case example by Journée et al. [12] A lower variability between measurements could help decrease the number of false positive warnings [15, 16].

When mTc-MEPs are monitored, the responsible neurophysiologist should ideally select a stimulus location and values of the stimulation parameters that generate optimal mTc-MEPs. In addition to the stimulation voltage, other stimulation parameters that can be varied include: stimulus duration, inter-stimulus interval, and number of pulses per train. Although multiple studies have tried to determine the optimal values of these stimulation parameters for eliciting mTc-MEPs, few studies have investigated the optimal stimulation parameters for performing supramaximal stimulation [12, 14, 17]. However, the feasibility of supramaximal stimulation for different stimulation parameters and the optimal stimulation parameters for performing supramaximal stimulation have not been vigorously investigated.

Therefore, the aim of this retrospective study was to investigate the feasibility of supramaximal stimulation for different stimulation parameters, and the optimal stimulation parameters, consisting of pulse duration and ISI, for performing supramaximal stimulation in patients undergoing scoliosis correction surgery.

## Materials and methods

### Study design

This is a retrospective observational study. Since the data analyzed in this study were collected during routine clinical care, the ethical committee of University Medical Center Groningen (UMCG) waived the requirement for full ethical committee review in accordance with the terms of the Dutch Act on Medical Research on Human Subjects (Wet Medisch-Wetenschappelijk Onderzoek, or ‘WMO’).

### Patients

The study data were collected from May 2018 until May 2019. Forty-seven consecutive patients undergoing scoliosis correction surgery with IONM were included.

### mTc-MEP monitoring

Intraoperative mTc-MEP monitoring was performed using a constant-voltage stimulator (NIM-Eclipse E4 IONM system, Medtronic BV, The Netherlands). Motor evoked potentials were measured using biphasic transcranial electrical stimulation [18]. For transcranial electrical stimulation, corkscrew electrodes (Medtronic, Xomed, Jacksonville, FL) were placed at location Cpl1-Cpl2 (1 cm posteriorly, 1 cm laterally from C1 resp C2) altered from the international 10–20 EEG System. Motor evoked potentials were recorded using surface electrodes (20 × 27 mm, adhesive surface pad electrodes, Medtronic, Xomed, Jacksonville, FL) from the left and right tibialis anterior muscle (TA), and the left and right abductor hallucis muscle (AH).

After anesthetic induction but before incision, mTc-MEPs were measured using two different settings. Setting 1 consisted of a pulse duration of 0.075ms and an ISI 1.5ms, while setting 2 consisted of a pulse duration of 0.300ms and an ISI 3ms. The maximum current output for was ± 1000mA for setting 1 and ± 180mA for setting 2, which are within the IEC safety limits [19]. The stimulation parameters are shown in Table 1.

**Table 1:** Stimulation parameters for the two different stimulation settings

	Setting 1	Setting 2
Stimulation location	Cpl1-Cpl2	Cpl1-Cpl2
Number of pulses	5	5
Pulse duration	0.075ms	0.300ms
Interstimulus interval	1.5ms	3.0ms
Maximum current output	1000mA	180mA

### Feasibility of supramaximal stimulation

For both setting 1 and setting 2, the feasibility of supramaximal stimulation was evaluated. To assess whether supramaximal stimulation was feasible, voltage intensity curves were produced by increasing the voltage in predefined steps of 10-20 V. Voltage intensity curves were determined per patient for both stimulation settings, and all four muscles separately (AH left and right, TA left and right). The mTc-MEP amplitudes were log-transformed and fitted to Sigmoidal-Boltzmann curves using GraphPad Prism (version 8.4.2, GraphPad Software, San Diego, California USA).

Two independent researchers (SED, GD) subsequently evaluated all curves to determine if supramaximal stimulation was achieved. Supramaximal stimulation was considered to have been achieved if the graph displayed a typical sigmoidal pattern with a plateau at the end of the curve.

**Fig. 1:**
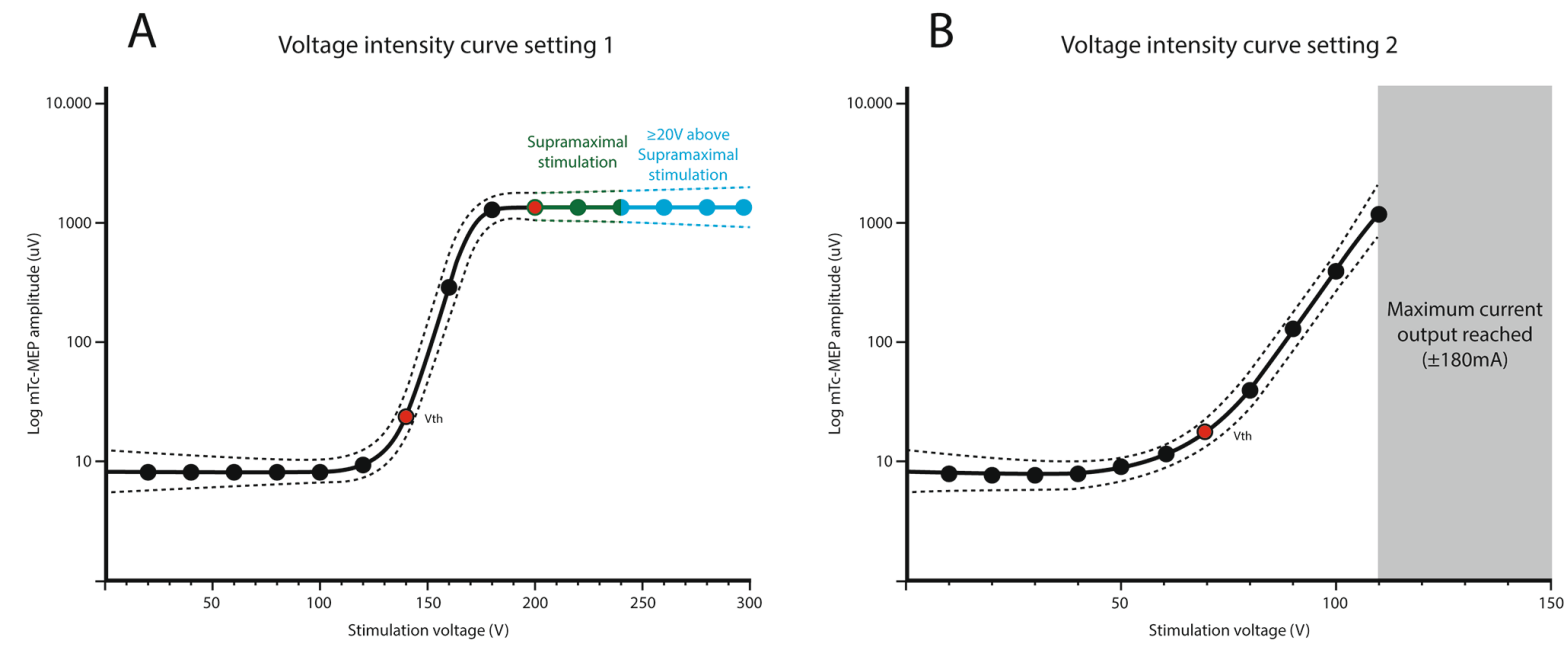
Example Sigmoidal Boltzmann curves for setting 1 (A) and setting 2 (B) The x-axis denotes the stimulation voltage used for eliciting the mTc-MEP amplitude. The y-axis denotes the logarithm of the mTc-MEP amplitude in µV. The graphs are altered from real data (**A**) supramaximal stimulation was achieved for setting 1 since a plateau was reached (green). In blue, the voltage intensities with the possibility to increase the voltage intensity with ≥ 20 V is shown The red dots represent the voltage motor threshold and the voltage intensity at which supramaximal stimulation was achieved (the second log-amplitude of the plateau). (**B**) Supramaximal stimulation was not reached for setting 2. It was not possible to increase the voltage above 110 V since the maximum current output was reached. The voltage intensity at which the maximum current output was reached differed between patients *Vth* Voltage threshold

The voltage intensity at which supramaximal stimulation was achieved was defined as the voltage necessary for the second log-amplitude on this plateau, in which it was verified that the log-amplitudes did not increase with higher voltage intensities **(**Fig. 1 A**)**.

Supramaximal stimulation might not be feasible for certain stimulation settings, considering the maximal stimulator output of the equipment used for measuring mTc-MEPs, due to the safety limit according to the International Electrotechnical Commission (IEC), which is 50 mJ through 1 kΩ resistance [19]. In Fig. 1B an example is shown in which supramaximal stimulation was not achieved for setting 2 since the maximum current output was reached.

The interobserver variability was calculated using Cohens Kappa. The disagreements were resolved by discussion between the two authors (SED, GD).

Due to the retrospective design of the study, in some patients, supramaximal stimulation was not achieved with one of the stimulation settings, although the maximal current output of the equipment was not yet reached. These patients were excluded from the analysis, as the possibility that supramaximal stimulation could have been achieved cannot be excluded.

### mTc-MEP parameters

After evaluating if supramaximal stimulation was achieved, three mTc-MEP parameters were considered of importance; (1) elicitability of muscles, (2) amplitude, and (3) if supramaximal stimulation was achieved with ≥ 20 V below maximum output to be able to address for anesthetic events. These three parameters were compared for both settings in all patients in which supramaximal stimulation was achieved.

Thereafter, we determined the optimal settings, taking into consideration whether supramaximal stimulation was achieved ánd the three abovementioned parameters together.

#### Number of elicitable muscles

A muscle was considered elicitable if a reproducible mTc-MEP amplitude could be observed at a display gain of 50µV. The elicitability of the TA muscles left and right and AH muscles left and right was evaluated after induction but before incision in all patients in which supramaximal stimulation was achieved for both settings separately.

#### mTc-MEP amplitude

The first mTc-MEP amplitudes of the TA and AH muscles that were measured after reaching supramaximal stimulation, were collected for both settings in all patients. The amplitudes were compared between setting 1 and 2.

#### Supramaximal stimulation achieved with ≥ 20 V below maximum output

During mTc-MEP spinal cord monitoring, the stimulation voltage or current should ideally not be at the maximum of the equipment used. Should higher anesthetic drug doses, anesthetic fade, or significant blood pressure decreases cause reductions in mTc-MEP amplitudes then it is desirable to increase the stimulation voltage or current [20, 21]. Therefore, we evaluated if there was the possibility to increase the stimulation voltage with ≥ 20 V below maximum output to address for these possible anesthetic events. The values of the voltage intensities and the corresponding current outputs at which supramaximal stimulation was achieved for all four muscles were collected. Thereafter, it was evaluated if it was possible to increase the voltage intensity with ≥ 20 V without reaching the maximum current output of our equipment.

#### Setting selection

Which stimulation setting would be the optimal setting according to all abovementioned parameters (feasibility, elicitability, amplitude and if supramaximal stimulation was achieved with ≥ 20 V below maximum output) was assessed retrospectively. First it was evaluated if supramaximal stimulation was achieved in all four muscles (AH left and right, TA left and right). If with one setting supramaximal stimulation was not achieved in one or more muscles for which the maximum current output had been reached, then the other setting was regarded as the preferred setting for mTc-MEP monitoring (if supramaximal stimulation was achieved with the other setting).

Thereafter, the number of muscles (AH left and right and TA left and right) in which an mTc-MEP could be elicited were considered. If a stimulation setting provided more elicitable muscles suitable for monitoring, then it was considered the preferred setting. Thirdly, the mTc-MEP amplitudes were compared between both settings. If with one setting the amplitudes were higher in at least 3 muscles, then that setting was considered preferable.

Lastly, we determined whether an increase of ≥ 20 V above that needed for supramaximal stimulation would cause the maximal current output of the equipment to be reached. If the maximal current output was reached for one setting, and was possible with the other, then the other setting was considered the optimal setting. After consideration of all parameters, the preferred stimulation setting was chosen.

### Interstimulus interval

Using a pulse duration of 0.075ms, the ISI that provided the highest amplitude for each of the AH and TA muscles was determined per patient. ISI’s of 1ms, 1.25ms, 1.5ms, 2ms, 3ms, and 4ms were used.

### Anesthesia

All patients underwent total intravenous anesthesia with propofol and remifentanil or sufentanil. Nondepolarizing muscle relaxants were administered judiciously during the induction of anesthesia to facilitate endotracheal intubation while not impeding the intraoperative detection of muscle responses. No other drugs which could interfere with neuromonitoring were administered before or during the measurements. Hemodynamic and respiratory parameters remained within the accepted physiological range in all patients.

### Statistical analyses

The mean and standard deviation (SD) were calculated for the baseline patient characteristics.

To determine if the amplitude of the TA and AH muscles differed significantly between both settings, the most parsimonious linear regression model was identified by testing whether setting (setting 1 vs. setting 2), side (left vs. right), and their interaction significantly improved model fit (p < 0.05) using sequential likelihood ratio tests (ANOVA function in R, R core team 2018, version 3.5.1). Model diagnostics were then performed on the most parsimonious model.

After developing the simple linear regression models, plots of the residuals were examined to determine if they were normally distributed. If they were not normally distributed, the variable was log-transformed using the natural logarithm.

The mean and SD or median and interquartile range (IQR) were calculated for the voltage intensities, current outputs and delivered charge from the last pulse of the train of pulses at which supramaximal stimulation was achieved, depending on whether the data was normally distributed or not. Differences in charge between both settings were analyzed using the Wilcoxon signed rank test.

## Results

### Patients

From the 47 consecutive patients, 9 (19.15%) were excluded. In four out of the nine excluded patients, measurements were performed with a pulse duration of 0.5ms instead of 0.3ms. One patient underwent surgery twice, therefore the data of the second operation was excluded to avoid clustering of data. In three patients, supramaximal stimulation was not achieved although the subsequent analyses showed that there had still been the possibility to increase the voltage without reaching the maximal current output. These three patients were therefore excluded from the analysis. The last patient was excluded since all four muscles (AH left/right, TA left/right) were not elicitable for both settings. Characteristics of the 38 included patients are listed in Table 2.

**Table 2:** Patient characteristics

	Patients (n = 38)
Age at surgery (Mean ± SD years)	16.83 (3.7)
Female N (%)	30 (78.9)
Idiopathic scoliosis N (%)	30 (78.9)
Syndromic scoliosis N (%)	7 (18.4)
Neurofibromatosis Type 1	2 (5.3)
Phelan-Mc Dermid Syndrome	1 (2.6)
Smith Magenis Syndrome	1 (2.6)
Chromosome 7 deletion	1 (2.6)
Marfan Syndrome	1 (2.6)
HERC1 mutation	1 (2.6)
Congenital scoliosis N (%)	1 (2.6)
Pre-operative motor weakness of the legs N (%)	2 (5.3)
Surgery time in minutes (SD)	314.3 (60.7)
Duration of hospitalization in days (SD)	7.8 (1.8)

*N* number, *SD* standard deviation

### Feasibility of supramaximal stimulation

In total 328 mTc-MEP voltage intensity curves were produced (41 patients, two sides, two muscles, two stimulation settings). Five muscles were not elicitable. Therefore, 323 curves (98.48%) were evaluated. The scores per researcher are shown in **supplementary table A**. Cohen’s Kappa was 82.54% which is considered ‘almost perfect’.

Supramaximal stimulation was achieved in all patients (100.00%) when stimulation was performed with setting 1. For setting 2, supramaximal stimulation was not achieved in 23 muscles in 12 patients (31.58%) out of 38 patients. The number of patients in which mTc-MEPs were elicitable and the number of patients in which supramaximal was achieved for both setting 1 and 2 are shown per muscle in Table 3.

**Table 3:** Number of elicitable muscles and feasibility of supramaximal stimulation for mTc-MEP monitoring of the AH left and right and the TA left and right

Muscle	N elicitable muscles	Setting 1	N elicitable muscles	Setting 2
AH left	37 (97.37%)	37 (100.00%)	37 (97.37%)	29 (78.38%)
AH right	38 (100.00%)	38 (100.00%)	38 (100.00%)	31 (81.58%)
TA left	38 (100.00%)	38 (100.00%)	36 (94.74%)	31 (86.11%)
TA right	38 (100.00%)	38 (100.00%)	37 (97.37%)	34 (91.89%)

*AH* abductor hallucis muscle, *TA* tibialis anterior muscle.

### mTc-MEP parameters

#### Number of elicitable muscles

Of the 38 patients in whom supramaximal stimulation was achieved when stimulating with setting 1, in 37 patients (97.37%) all four muscles were elicitable. In 25 (96.415%) out of the 26 patients in which supramaximal stimulation was achieved when stimulating with setting 2, all four muscles were elicitable. In this one patient in which not all four muscles were elicitable, the AH left and TA left muscle were not elicitable for setting 2, and in the same patient only the AH left was not elicitable for setting 1.

#### mTc-MEP amplitude

For all 26 patients in whom supramaximal stimulation was achieved for both settings, the amplitude was compared using linear regression analysis. The amplitude was not statistically different between setting 1 and 2 for the AH muscles (F(1, 100) = 0.70, p = 0.40) or for the left or right side of the AH (F(1, 100) < 0.01, p = 0.97).

For the TA muscles, the amplitude was also not statistically different between setting 1 and 2 (F(1, 101) < 0.01, p = 0.91) or for the left or right side of the TA (F(1, 101) = 0.48, p = 0.49).

**Fig. 2:**
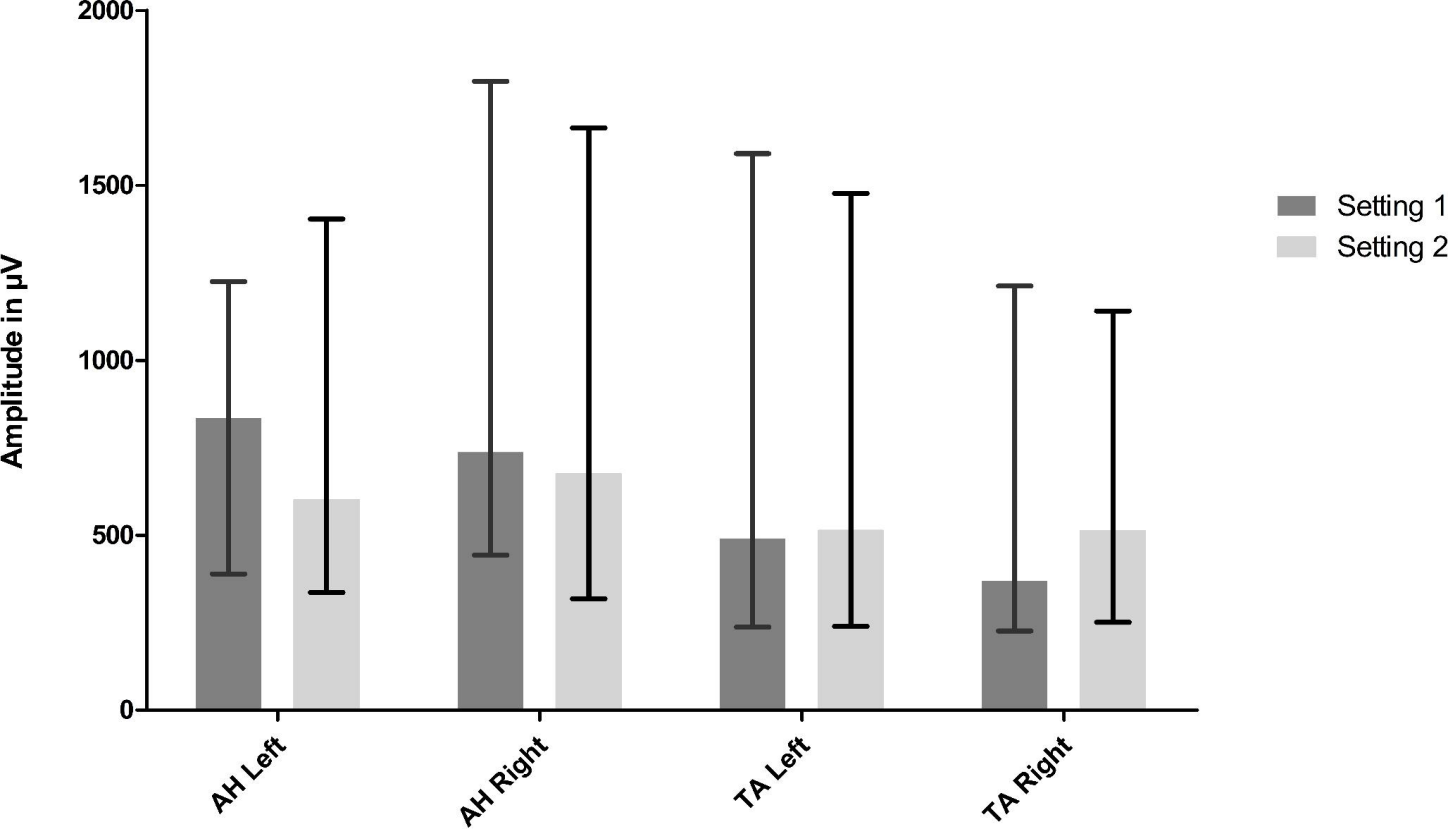
Median amplitudes per muscle for setting 1 and setting 2 The x-axis denotes the different muscles used for mTc-MEP monitoring, and the y-axis denotes the median amplitude in µV. The median intraoperative mTc-MEP amplitudes for setting 1 (dark grey bars) and setting 2 (light grey bars) are presented of 25 patients for the AH left and 26 patients for the AH right, TA left and TA right. The whiskers represent the interquartile range (Q1-Q3). *AH* abductor hallucis muscle, *TA* tibialis anterior muscle

The median amplitudes and IQR per muscle and per setting can be found in Fig. 2.

#### Supramaximal stimulation achieved with ≥ 20 V below maximum output

In all 38 patients in whom supramaximal stimulation was achieved using stimulation setting 1, the voltage could be increased with ≥ 20 V above that required for supramaximal stimulation without reaching the maximal current output of the equipment. For setting 1, the mean voltage necessary to obtain supramaximal stimulation was 279.21 V (SD 69.06) and the median current was 393.50mA (IQR 348.25mA – 511.75mA).

In 10 (38.46%) out of the 26 patients in whom supramaximal stimulation was achieved using stimulation setting 2, the voltage could be increased with ≥ 20 V above that required for supramaximal stimulation without reaching the maximal current output. Of the 26 patients in whom supramaximal stimulation was achieved, the mean voltage was 102.12 V (SD 14.01) and the median current was 158.00mA (IQR 144.75mA – 178.00mA). The median charge of the last pulse of the train of five pulses after supramaximal stimulation was 29.5µC for setting 1 (n = 38) and 47.4µC for setting 2 (n = 26). The delivered charge was significantly lower for setting 1 (p < 0.001) as evidenced by the Wilcoxon signed rank test.

### Setting selection

#### Feasibility of supramaximal stimulation

**Fig. 3:**
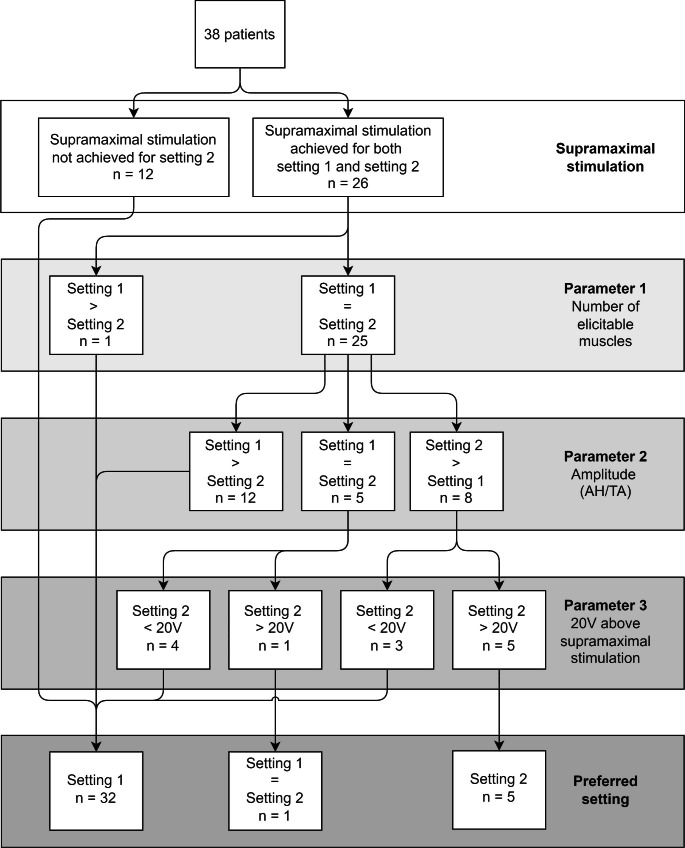
Overview selection criteria for choosing either setting 1 or setting 2 for monitoring mTc-MEPs using supramaximal stimulation *AH* abductor hallucis muscle, *TA* tibialis anterior muscle

In 12 out of 38 patients (31.58%) in whom supramaximal stimulation was not achieved in all four muscles when stimulating with setting 2, the maximal current output was already reached. Since, supramaximal stimulation was achieved when stimulating with setting 1, it was considered to be the preferred stimulation setting in these 12 patients (Fig. 3).

#### Number of elicitable muscles

In one patient (3.85%), the AH left and the TA left muscle were not elicitable for setting 2, and in the same patient, only the AH left was not elicitable for setting 1. Therefore, for this patient, setting 1 was the preferred setting.

#### mTc-MEP amplitude

In 12 patients (48.00%) out of the remaining 25 patients, amplitudes were higher in at least 3 of the TA and AH muscles when stimulation with setting 1. In 5 patients (20.00%), the number of muscles that had the highest mTc-MEP amplitudes were equal. In 8 patients (32.00%) setting 2 was preferred considering that the mTc-MEP amplitudes were higher in at least 3 muscles than when stimulating with setting 1.

#### Supramaximal stimulation achieved with ≥ 20 V below maximum output

For setting 1 it was possible in all 38 patients (100.0%) to increase the voltage with ≥ 20 V above that required for supramaximal stimulation.

In 5 (62.50%) of the 8 patients in whom setting 2 provided higher amplitudes in at least 3 muscles when compared to setting 1, the voltage intensity could be increased with ≥ 20 V above supramaximal stimulation for setting 2. In the remaining 3 patients (37.50%) this was not possible for setting 2. In 1 (20.00%) of the 5 patients (18.52%), in whom the number of muscles that had the highest mTc-MEP amplitudes were equal, the voltage could be increased with ≥ 20 V above supramaximal stimulation for setting 2. In the remaining 4 patients (80.00%) this was not possible.

#### Optimal setting

In 32 patients (84.21%) setting 1 was preferred when compared to setting 2 considering the abovementioned parameters. In 1 patient (2.63%), the settings were equally preferable and in 5 patients (13.16%) setting 2 was preferred.

### Interstimulus interval

**Fig. 4:**
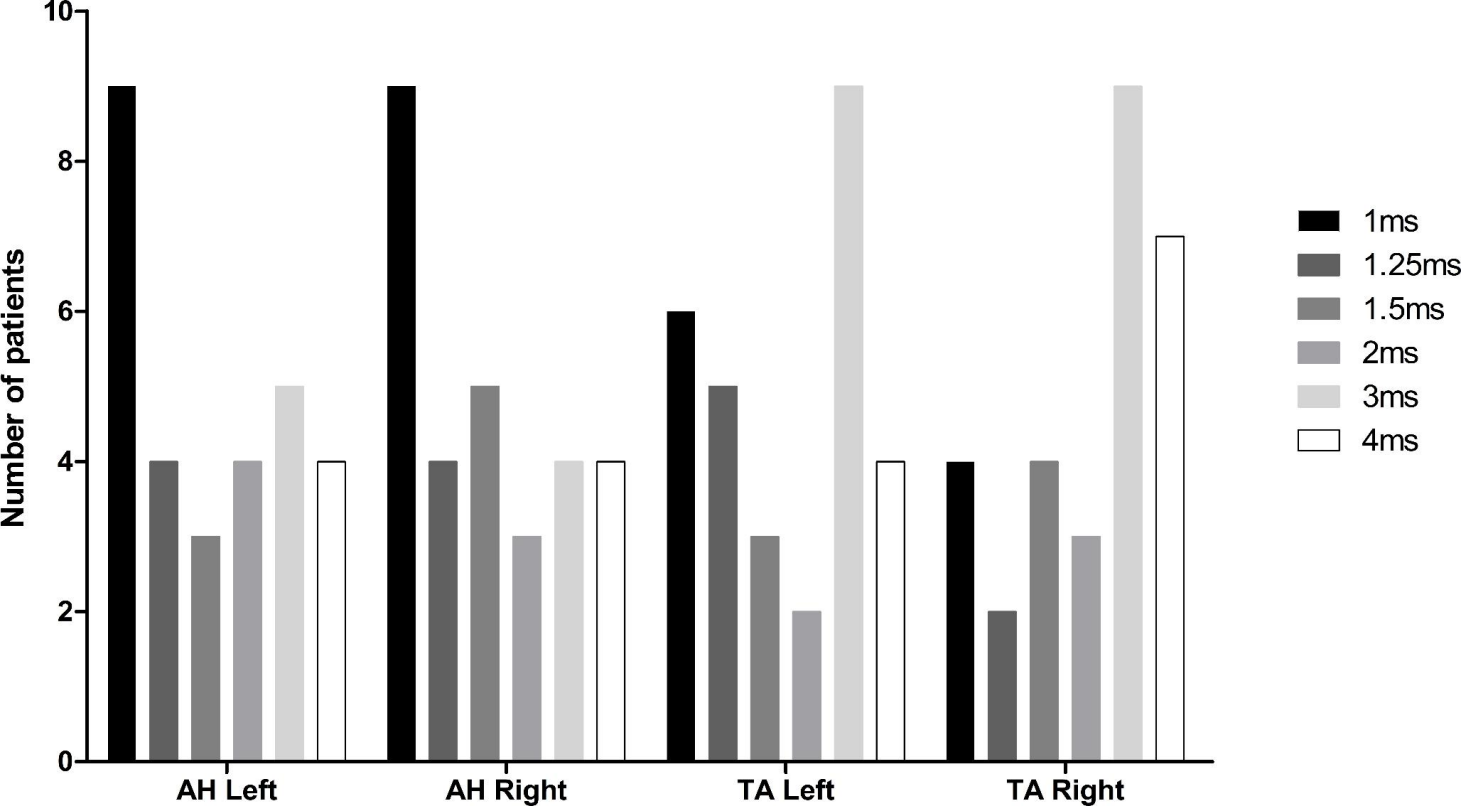
Distribution of optimal ISI when measuring mTc-MEPs using supramaximal stimulation with a pulse duration of 0.075ms (n = 29) The x-axis denotes the different muscles used for mTc-MEP monitoring and the y-axis denotes the number of patients. The optimal ISI’s ranging from 1ms to 4ms are presented by the different colored bars for the AH left, AH right, TA left and TA right separately *AH* abductor hallucis muscle, *TA* tibialis anterior muscle

In 9 (23.68%) out of 38 patients, not all ISI’s were used and they were therefore excluded from this analysis. In Fig. 4, the number of muscles of the optimal ISI’s can be observed. For both the AH left and right, an ISI of 1ms provided the highest amplitude in most patients (n = 9, 31.03%) for both AH left and right). For the TA left and right, an ISI of 3ms provided the highest amplitude in most patients (n = 9, 31.03%) for both TA left and right). However, no clear optimal ISI can be observed per muscle, and were often even different between muscles within a patient.

## Discussion

In this study we investigated the feasibility of supramaximal stimulation for different stimulation parameters, as well as the optimal stimulation parameters, consisting of pulse duration and ISI, for performing supramaximal stimulation in patients undergoing scoliosis correction surgery.

Firstly, our study showed that supramaximal stimulation is most feasible when stimulating with a relatively short pulse duration (0.075ms) when compared to a relatively long pulse duration (0.300ms). Secondly, for selection of optimal stimulation parameters for performing supramaximal stimulation, the maximum stimulator output should be considered, since ideally there has to be the possibility to increase stimulation voltage or current to be able to compensate for anesthetic events. Lastly, our study showed that when monitoring mTc-MEPs using supramaximal stimulation with a pulse duration of 0.075ms, there is broad inter-individual variation in the optimal ISI. This suggests that the optimal ISI should be determined in a per patient basis.

Our study evaluated sigmoidal curves to determine whether supramaximal stimulation was feasible for two different stimulation settings. The mTc-MEP amplitudes of the voltage intensity curves were log-transformed since changes in the amplitude are usually considered in a relative context and described by a ratio rather than by an absolute difference [12]. Two independent researchers (SED, GD) evaluated all sigmoidal curves to determine whether supramaximal stimulation was achieved. We then assessed the agreement between their evaluations and found that the Cohen’s Kappa was 82.54% which is considered ‘almost perfect’ agreement. This high Cohen’s Kappa implies that this is a good method for assessing if supramaximal stimulation was achieved or not.

It is clinically relevant to know if supramaximal stimulation was achieved in all muscles, since this has an influence on the interpretation of the warning criteria. If mTc-MEP monitoring is performed using submaximal stimulation, the potentially large inter-trial variability between mTc-MEP measurements can give false positive warnings. The difference in inter-trial variability when submaximal stimulation and supramaximal stimulation are used requires further investigation, since up till now it has only been shown in one case report [12].

Unfortunately, the equipment used in this study cannot display voltage intensity curves during mTc-MEP monitoring. Therefore, in clinical practice, supramaximal stimulation was assumed if, by visual inspection, the mTc-MEP amplitude did not increase anymore after increasing voltage intensity.

Supramaximal stimulation was feasible in all patients, when stimulating with setting 1. We found that with setting 2, supramaximal stimulation was not achieved in 12 patients (31.58%), since the maximal current output of the stimulator had been reached. Therefore, with longer pulse durations, supramaximal stimulation might not always be possible depending on the limits of the equipment used for mTc-MEP monitoring. If this is also true for constant current stimulators needs to be further explored.

For the patients in whom supramaximal stimulation was achieved for both settings, the elicitability of mTc-MEPs was similar when performing supramaximal stimulation. There was only one patient in whom fewer muscles were elicitable with setting 2 when compared to setting 1.

There were more patients that had higher mTc-MEP amplitudes in at least three of the four recorded muscles when stimulating with setting 1. However, the amplitudes did not differ significantly between the two settings.

When stimulation with setting 2, in most patients, supramaximal stimulation was only achieved with a current at or close to the maximal current output, as can be deduced from the high median current of 162.50mA (IQR 145.00mA – 178.75mA). During surgery, and especially during long operations such as scoliosis correction, it should ideally be possible to increase the voltage intensity to counteract the potential influence of the effects of higher anesthetic drug doses, anesthetic fade, and significant blood pressure decreases on mTc-MEP amplitudes [20–22]. Although, the ≥ 20 V cut-off value chosen in this study is arbitrary, in our experience, it was sufficient to counteract for anesthetic events.

Another advantage of stimulating with setting 1 was that the median charge from the last pulse of the train of pulses was significantly lower (p < 0.001) when compared to setting 2. One could argue that stimulating with higher voltages and a relatively short pulse duration, compared to stimulating with lower voltages and a relatively long pulse duration, may result in similar amounts of delivered charge. Theoretically, shorter ISIs also result in higher amounts of delivered charge per train of pulses. Our results suggest that setting 1 can achieve supramaximal stimulation of mTc-MEPs more efficiently than setting 2. Unfortunately, the ISIs were not included in the calculations since the precise delivered charge per train was not available from the equipment used in this study.

As mentioned above there was significant inter-individual variability in optimal ISI, and also within patient variability per muscle. Optimal ISI should therefore be determined per patient.

Published data concerning the optimal ISI for mTc-MEP monitoring are conflicting. In general, ISIs between 2 and 5ms are recommended [7, 23–27]. Using methodology similarly to that of our study, Hal et al. also found a bimodal distribution with a first peak at around 1ms, and the second peak at around 3ms for the tibialis anterior muscles. However, it could be argued that the short ISI’s of 1ms might result in stimulation within the absolute refractory period (ARP). The relationship between the ARP and the optimal ISI for providing the highest amplitude is not known. Novak et al. reported that the ARP is shorter (mean 0.82ms) with supramaximal stimulation than with submaximal stimulation (mean 1.47ms) [28]. This might explain why shorter ISI’s than 2ms can be the optimal ISI for supramaximal stimulation, which is contradictory to previous literature [7, 23–27].

The difference between the optimal ISI’s of the TA and AH muscles might be explained by differences of the motor unit properties of the different muscles [28]. Further research is necessary to better understand how mTc-MEP stimulation parameters can be optimized per muscle and how the different motor unit properties of different muscles play a role in mTc-MEP stimulation. Taken together, our data and that of others suggests that optimal ISI will depend on whether supramaximal stimulation or submaximal stimulation is used to elicit mTc-MEPs, and furthermore there is inter-patient variability and within-patient (per muscle) variability in optimal ISI for obtaining the highest amplitude. An individualized optimization of the ISI is therefore still recommended [14].

In our clinical practice, we choose to first obtain supramaximal stimulation, and thereafter optimize the ISI (1ms, 1.25ms, 1.5ms, 2.0ms, 3.0ms and 4.0ms) and choose the ISI that provided the highest mTc-MEP amplitudes in the muscles of most interest.

Although this study is one of the most extensive studies to determine the feasibility of supramaximal stimulation for different stimulation parameters, and the optimal stimulation parameters, consisting of pulse duration and ISI, it has limitations.

Firstly, although we investigated two different stimulation settings, there are still multiple different settings of interest that have to be investigated to get more insight into the optimal setting parameters for supramaximal stimulation. However, due to time constraints in the operating theater and patient safety concerns, at the time of data collection we routinely only used two sets of settings. By understanding more about the range of the different stimulation parameters for optimization of supramaximal stimulation, a more targeted set-up for the baseline measurements can be achieved in the limited time available in the operating room.

Another limitation of supramaximal stimulation might be that it may not be suitable for all types of surgery, since with higher voltage or current stimulation, there will be more patient movement. Supramaximal stimulation might, for example, not be feasible for neurosurgical patients in whom the head is secured in a Mayfield skull clamp.

## Conclusion

mTc-MEP monitoring using supramaximal stimulation is most feasible when stimulating with a relatively short pulse duration (0.075ms) since with a relatively longer pulse duration (0.300ms) the maximum stimulator output can be reached before supramaximal stimulation is achieved or there is no possibility to increase the voltage intensity to counteract for anesthetic events. Therefore, we recommend using setting 1 when monitoring mTc-MEPs with supramaximal stimulation, after which an individualized ISI optimization can be performed. Moreover, when using supramaximal stimulation, short ISI’s (i.e. 1ms or 1.5ms) can be the optimal ISI for obtaining the highest mTc-MEP amplitude.

## Electronic supplementary material

Below is the link to the electronic supplementary material.

Supplementary Material 1

## Abbreviations

mTc-MEP: muscle evoked transcranial electrical stimulation motor evoked potential
ISI: interstimulus interval
AH: abductor hallucis muscle
TA: tibialis anterior muscle
IONM: intraoperative neurophysiological monitoring
SSEP: somatosensory evoked potential
IQR: interquartile range
IEC: International Electrotechnical Commission
