# Supplementary material for: Feasibility and optimal choice of stimulation parameters for supramaximal stimulation of motor evoked potentials
Source: J Clin Monit Comput. 2023 Jan 13;37(3):783–93. doi: 10.1007/s10877-022-00972-5 (PMC10175431; doi:10.1007/s10877-022-00972-5)
Supplement: Supplementary file 1 — Supplementary Material 1 [file 10877_2022_972_MOESM1_ESM.docx]

**Supplementary Table A**

**Table A.** Interobserver variability

|  |  | Researcher 1 | |  |
| --- | --- | --- | --- | --- |
| Researcher 2 |  | Supramaximal stimulation achieved N (%) | Supramaximal stimulation not achieved N (%) | Total N (%) |
|  | Supramaximal stimulation achieved N (%) | 298 (92.26%) | 5 (1.55%) | 303 (93.81%) |
|  | Supramaximal stimulation not achieved N (%) | 2 (0.62%) | 18 (5.57%) | 20 (6.19% |
|  | Total N (%) | 300 (92.88%) | 23 (7.12%) | 323 (100.00%) |
